# A self-correcting Agentic Graph RAG for clinical decision support in hepatology

**DOI:** 10.3389/fmed.2025.1716327

**Published:** 2025-12-16

**Authors:** Yalan Hu, Wenjie Xuan, Qingqing Zhou, Zhi Li, Ya Li, Jili Hu, Fang Fang

**Affiliations:** School of Medical Information Engineering, Anhui University of Chinese Medicine, Hefei, China

**Keywords:** Agentic Graph RAG, knowledge graph, self-correction, large language models, clinical decision support, hepatology

## Abstract

**Introduction:**

Clinical decision-making in hepatology is currently challenged by the rapid expansion of medical knowledge and the limitations of Large Language Models (LLMs), specifically their unreliability and tendency to hallucinate. Furthermore, standard Retrieval-Augmented Generation (RAG) paradigms often fail to effectively leverage complex medical knowledge structures.

**Methods:**

To address these issues, we propose an Agentic Graph RAG framework built upon a clinically-verified hepatology knowledge graph. Our approach utilizes a state-driven agentic system employing a self-correcting “retrieve-evaluate-refine” loop. Within this workflow, agents dynamically generate, semantically validate, assess, and iteratively optimize graph search strategies to construct a comprehensive and accurate context, which is then used by an LLM to generate reliable responses.

**Results:**

The framework was evaluated on a custom dataset of clinical questions. It significantly outperformed baseline models (including GPT-4, standard RAG, and Graph RAG) across all evaluation metrics. Specifically, our model achieved superior scores in faithfulness (0.94), context recall (0.92), and answer relevancy (0.91).

**Discussion:**

This agentic approach effectively mitigates LLM hallucinations and provides accurate, interpretable answers. These findings demonstrate the framework's potential as a robust, next-generation intelligent clinical decision support tool for hepatology.

## 1 Introduction

### 1.1 The evolving role of clinical decision support in hepatology

Clinical decision-making in hepatology is characterized by high stakes and significant complexity. Liver disease constitutes a major global public health concern, responsible for approximately 2 million deaths annually, or 4% of all global mortality (1). Amid an unprecedented expansion of medical knowledge, clinicians face a significant information processing burden. They must efficiently integrate patient-specific data with extensive public medical knowledge bases under tight time constraints to formulate optimal treatment plans (2). This substantial cognitive load underscores the urgent necessity for novel intelligent tools that can assist physicians in making precise and efficient clinical decisions (3), thereby enhancing the overall quality of healthcare delivery (4).

### 1.2 Limitations of existing AI-assisted clinical paradigms

Traditional Clinical Decision Support Systems (CDSS) have predominantly relied on rule-bases predefined by domain experts or conventional machine learning models (5, 6). While effective in specific contexts, their rigid logic impedes adaptation to rapidly evolving clinical guidelines (7) and limits their ability to flexibly interpret complex, natural language queries from physicians (8). More recently, the new generation of artificial intelligence, particularly Large Language Models (LLMs), has demonstrated exceptional capabilities in natural language processing (9, 10). However, the direct application of general-purpose LLMs in high-stakes clinical settings is fraught with the significant challenge of factual hallucinations, wherein the model may generate information that is plausible yet factually incorrect (11).

As a mitigation strategy, Retrieval-Augmented Generation (RAG), first proposed by Lewis et al. (12) and explored in medicine, grounds LLMs in external knowledge bases. Although RAG alleviates hallucinations (13, 14), standard implementations (our first baseline) retrieve unstructured text fragments (15), failing to capture the deep, logical relationships inherent in medical knowledge (16).

This limitation has driven the development of frameworks that integrate Knowledge Graphs (KGs) with RAG. Recent advances have shown significant promise: KG**-**RAG (17) and KRAGEN (18), for example, demonstrate effective methods for enhancing biomedical RAG with structured data. MedRAG (19) proposes a comprehensive RAG system for clinical medicine, while Zebra-Llama (20) highlights the critical need for grounding LLMs in specialized domains, such as hepatology. These approaches leverage KGs as a structured factual backbone. However, many basic Graph RAG implementations (our second baseline) still suffer from two critical gaps. They lack a mechanism to validate the generated graph queries, leading to the execution of “syntactically valid but semantically incorrect” queries that retrieve erroneous information. They lack a robust process to evaluate the sufficiency of the retrieved context, proceeding to answer generation with incomplete information. Crucially, for safe clinical deployment, an AI system must possess a reliable mechanism to evaluate information sufficiency and handle ambiguity, a feature missing in non-agentic architectures (21).

### 1.3 A Novel framework integrating structured knowledge with agentic reasoning

To overcome these limitations, this paper proposes and implements an innovative framework that achieves a deep fusion of a high-precision, domain-specific Knowledge Graph (KG) with an intelligent Agent capable of autonomous planning and reasoning (22). In this system, the KG serves as a verifiable factual foundation, while the Agent functions as an adaptive reasoning engine (22–24). We first constructed an expert-verified hepatology KG that precisely encodes deterministic medical knowledge (25–27), serving as a reliable factual anchor to mitigate hallucinations (28). Concurrently, using the LangGraph framework (29, 30), we designed a dynamic agentic workflow (31, 32). This agent autonomously translates complex clinical questions from natural language into precise, executable Cypher queries for the graph database (Text-to-Cypher) (33–35). Crucially, the agent first submits this query to a semantic validation module to ensure logical correctness before execution.

Upon retrieving the initial information, the agent enters a multi-layered decision module. This module employs self-consistency voting, dual-model cross-validation, and a non-LLM vector similarity failsafe mechanism to rigorously evaluate the sufficiency of the context (36). If the context is deemed insufficient, the agent activates a deterministic strategy optimizer. This module programmatically modifies the search strategy and then re-executes the original, validated query. This “validate-execute-evaluate” loop iterates until a satisfactory context is assembled.

The synergy between these components is crucial: the KG provides the agent with factual knowledge, while the agent makes the KG's complex knowledge accessible via natural language. This architecture effectively bridges the gap between complex structured data and the practical needs of clinicians. The primary contributions of this research are 3-fold:

1 Construction of a High-Quality Hepatology Knowledge Graph: We developed a domain-specific KG for liver disease containing 12,192 entities and 28,770 relationships, verified by clinical experts. This serves as a high-quality, structured factual foundation to mitigate hallucinations in LLMs.

2 Proposal of a Novel Agentic Graph RAG Framework with Self-Correction: We introduced a deterministic, multi-stage self-correction loop to ensure high reliability. First, we implement a dual-model, self-consistency evaluation, wherein two independent LLMs each cast three votes to robustly assess the context's sufficiency. In case of model disagreement, a semantic relevance fallback mechanism provides a quantified, non-LLM adjudication based on vector similarity. Finally, we employ a deterministic, algorithmic-level strategy optimizer that algorithmically optimizes the search strategy based on evaluation metrics, ensuring a predictable and efficient optimization loop.

3 Experimental Validation of the Framework's Efficacy: Our experiments demonstrate that the synergy between the self-correcting agent and the KG significantly outperforms baseline methods—including general LLMs and traditional RAG—in terms of answer accuracy, completeness, and faithfulness across a range of clinical questions involving simple facts, multi-step reasoning, and ambiguity.

## 2 Materials and methods

### 2.1 Overall architecture of the Agentic Graph RAG framework

**Figure 1:**
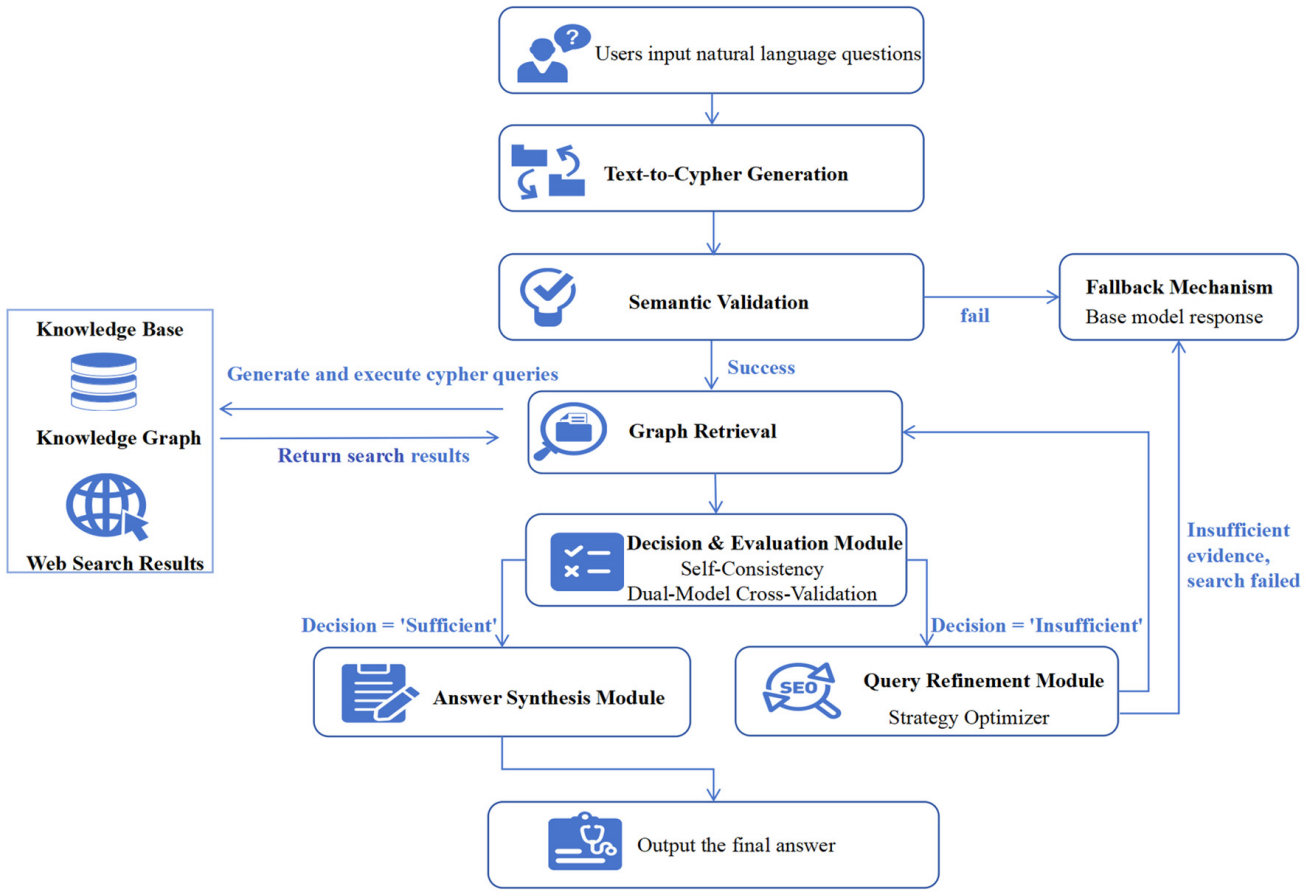
The overall architecture of the system. This figure shows the system's overall architecture, detailing how a user's query is processed through retrieval, evaluation, an optional refinement loop, and final answer synthesis.

The Agentic Graph RAG framework proposed in this study is an intelligent system designed to simulate the deliberative reasoning process of hepatology experts. This is achieved through the collaboration between a comprehensive liver disease knowledge graph and a self-correcting intelligent agent. The overall architecture of the system, depicted in Figure 1, illustrates the deep integration of a static knowledge base (the knowledge graph) with a dynamic reasoning engine (the agent) to accurately address complex clinical questions.

The liver disease knowledge graph serves as the factual foundation of the system. It precisely encodes complex medical concepts and their interrelationships as entities and relations, providing a solid factual anchor for the agent's reasoning processes. This component is fundamental to ensuring the accuracy of the final output.

The self-correcting agent, which operates based on a dynamic workflow, is the system's reasoning engine. It is designed to interpret a user's natural language query, autonomously plan and generate corresponding graph queries, and critically evaluate the retrieved information. If the initial information is insufficient, the agent initiates a self-correction loop to refine its query. Finally, it synthesizes the verified factual evidence into a coherent and professional natural language response. Within this framework, the agent proactively queries the knowledge graph to acquire facts that constrain its internal reasoning and generation processes, thereby effectively preventing factual hallucinations.

### 2.2 Construction of the liver disease knowledge graph

**Figure 2:**
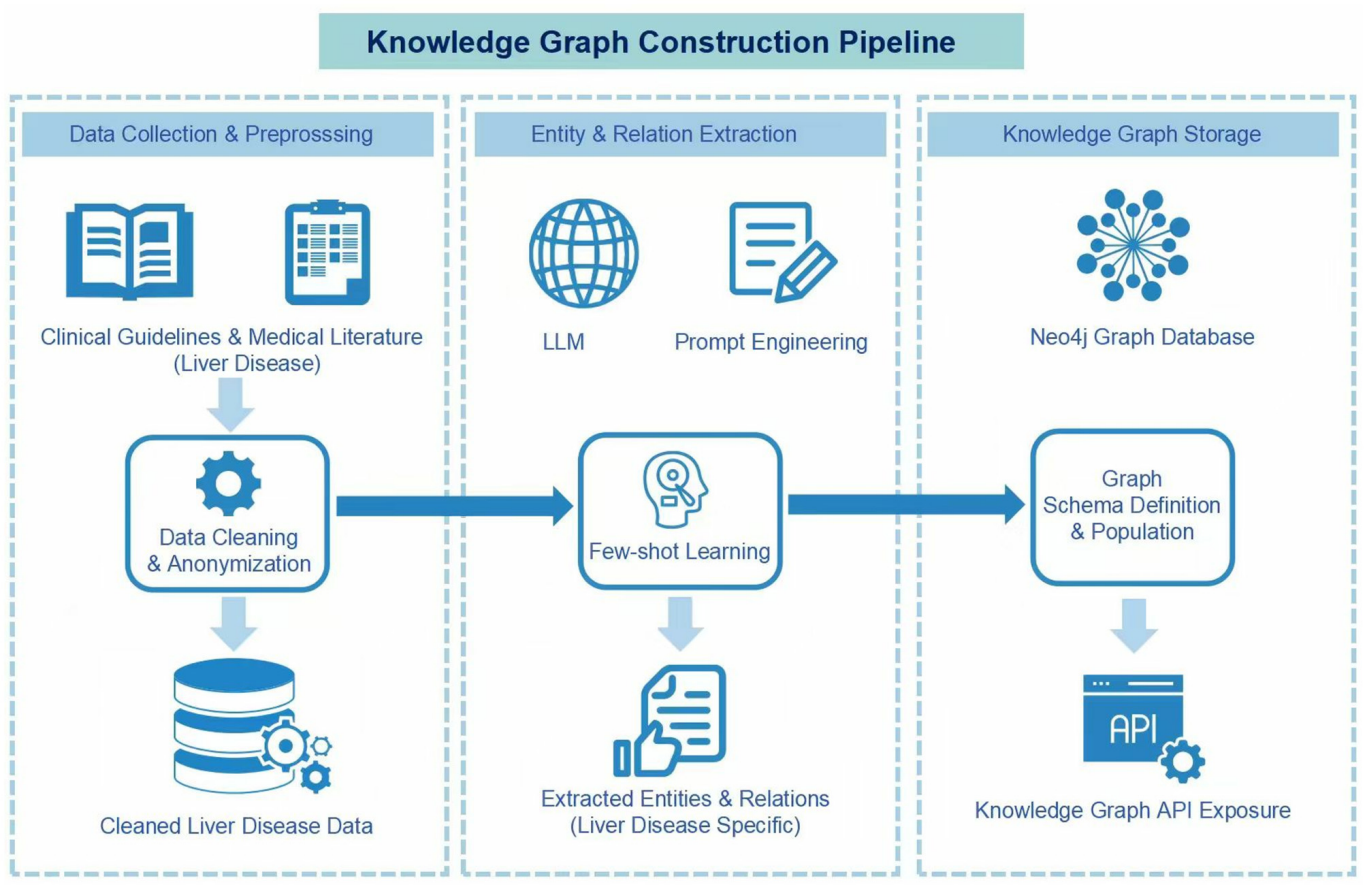
Knowledge graph construction pipeline. This figure outlines the three-stage pipeline for building the knowledge graph, which involves data collection, knowledge extraction using LLMs with expert review, and storage in a database.

To establish a high-precision and trustworthy knowledge base for the downstream reasoning agent, we implemented an enhanced semi-automated construction pipeline. This process deprecates simple co-occurrence methods in favor of an attribute-rich property graph model that combines LLM-based extraction with rigorous expert verification. The process consists of three primary stages: data collection and preprocessing, attribute-rich schema design, and evidence-based knowledge extraction, as illustrated in Figure 2.

#### 2.2.1 Data sourcing and pre-processing

To ensure the accuracy and currency of the knowledge, we sourced data exclusively from authoritative clinical literature. Using PubMed (https://pubmed.ncbi.nlm.nih.gov/) as the primary search platform, we developed a systematic search strategy and curated a corpus of 53 highly influential clinical practice guidelines on liver disease published within the last 15 years. The raw PDF documents were converted to plain text suitable for NLP using the PyMuPDF library to automatically extract the core textual content (37). Subsequently, we performed a series of text cleaning and normalization procedures, programmatically removing non-informational elements such as headers, footers, captions, and reference lists to minimize noise during knowledge extraction. To ensure the consistency and standardization of medical terminology, we leveraged the Metathesaurus of the Unified Medical Language System (UMLS). Key medical concepts identified in the text were mapped and aligned with standard entities via their Concept Unique Identifiers (CUIs), laying a robust foundation for the construction of a high-quality, unambiguous knowledge graph) (38).

#### 2.2.2 Attribute-rich knowledge graph schema design

This study employs a top-down, domain expert-driven strategy for schema construction to ensure both domain completeness and clinical utility. First, we pre-defined eight core entity categories designed to comprehensively cover the key informational dimensions within clinical guidelines. These categories include: Disease, Drug, Gene, Symptom/Sign, Examination/Test, Treatment, Etiology/Risk Factor, and Clinical Criteria. Each entity category is required to possess standardized attributes, including a UMLS ontology mapping and a normalized name. To capture clinically significant and quantifiable evidence-based associations, we pre-defined 11 relation types. The core RecommendsDrug relation, for instance, was designed to include attributes for Evidence Level, Recommendation Strength, and Drug Approval Status, as well as a dosing regimen specifying Dosage, Route of Administration, and Frequency. Furthermore, we introduced a weight attribute, designed to quantify the comprehensive confidence of a recommendation during subsequent aggregation phases.

This “evidence-driven” schema design enables us to structurally store complex relations, such as those describing pathophysiology and molecular biology. The final 8 entity and 11 relation definitions, confirmed via expert review, form the ontology of our knowledge graph. The entity types are enumerated in Table 1, and the relation types are detailed in Table 2.

**Table 1:** Entity types.

**Entity type**	**Description**
Disease	Refers to any condition that deviates from or interrupts normal function, typically characterized by specific signs and symptoms.
Drug	A chemical substance used for the diagnosis, cure, treatment, or prevention of disease.
Gene	The basic unit of genetic information, often associated with specific diseases or as a target for drugs.
Symptom/sign	Symptom: A subjective abnormality reported by the patient (e.g., pain, dizziness). Sign: An objective abnormality found upon examination (e.g., jaundice, hypertension).
Examination/test	Medical procedures or tests used to aid diagnosis, such as imaging (CT, MRI) or laboratory tests (blood work).
Treatment	Therapeutic methods other than drugs, such as surgery, radiotherapy, physical therapy, or lifestyle interventions.
Etiology/risk factor	Etiology: The direct cause of a disease (e.g., a virus). Risk Factor: A factor that increases the likelihood of developing a disease (e.g., smoking).
Clinical criteria	Specific rules, guidelines, or scoring systems used to diagnose a disease or guide treatment.

This table defines the 8 core entity types that constitute the liver disease knowledge graph.

**Table 2:** Relation types.

**Relationship**	**Description**	**Example connection**
RECOMMENDS_DRUG	Indicates that a specific drug is recommended for treating a disease.	Disease -> drug
HAS_ADVERSE_EFFECT	Indicates that a drug may cause an undesirable side effect (often a symptom).	Drug -> symptom/sign
ASSOCIATED_WITH_GENE	Indicates that a disease is linked to a specific gene's variation or expression.	Disease -> gene
TARGETS_GENE	Indicates that a drug's mechanism of action involves a specific gene or its product.	Drug -> gene
IS_CONTRAINDICATED_FOR	Indicates that a drug should not be used for a patient with a specific disease or condition.	Drug -> disease
REQUIRES_CRITERIA	Indicates that the diagnosis or staging of a disease depends on specific clinical criteria.	Disease -> clinical criteria
HAS_SYMPTOM	Indicates that a disease manifests with specific symptoms or signs.	Disease -> symptom/sign
IS_DIAGNOSED_BY	Indicates that a disease can be confirmed using a specific examination or test.	Disease -> examination/test
IS_CAUSED_BY	Indicates that a disease is caused by a specific etiology or risk factor.	Disease -> etiology/risk factor
RECOMMENDS_TREATMENT	Indicates that a specific non-drug treatment is recommended for a disease.	Disease -> treatment
PROGRESSES_TO	Indicates that one disease (or state) may evolve into another, often more severe, disease (or state).	Disease -> disease

This table details the 11 types of relationships used in the knowledge graph to connect entities.

#### 2.2.3 Knowledge extraction via a human-in-the-loop workflow

To ensure the precision and efficiency of knowledge extraction, this research employs a Human-in-the-loop (HITL) workflow, integrating automated Large Language Model (LLM) processing with manual expert review. First, during the automated processing stage, we segment the preprocessed text into semantically coherent chunks, accommodating the LLM's context window limitations. We then utilize GPT-3.5-Turbo, constrained by a meticulously designed system prompt. This prompt defines the model's role and includes the complete JSON Schema, directing the model to precisely identify the 8 predefined entity and 11 relation types from each chunk and output them in a strict JSON format. Second, in the knowledge fusion stage, the raw extractions undergo a rigorous fusion and quality control process. After merging all fragments, we perform hierarchical deduplication on entities, prioritizing the UMLS CUI as the unique identifier, falling back to a normalized name if the CUI is absent, and using the original text only as a last resort. This ensures conceptual uniqueness. For relations, we apply a distinct fusion strategy. For core, weighted relations like RecommendsDrug, we implemented an aggregation algorithm. When merging multiple relations for the same (Disease, Drug) pair, the system automatically calculates a mean weight and selects the strongest recommendation level, thereby intelligently synthesizing evidence from multiple sources. Finally, in the manual review stage, although the LLM exhibits powerful automated capabilities, all automatically extracted and fused entities and relations are submitted to hepatology domain experts for review and validation. This step is critical to ensure the utmost accuracy and authoritativeness of the knowledge for clinical application. The experts are tasked with correcting erroneous extractions and supplementing any omissions. This verification process can be formalized as shown in Equation 1:


$$\begin{array}{l} E_{final}=\left(E_{LLM}\backslash E_{remove}\right)\cup E_{add} \end{array}$$ (1)

Where *E*_*LLM*_ is the initial set of entities extracted by the LLM, *E*_*remove*_ is the set of erroneous or irrelevant entities identified by experts for removal, *E*_*add*_ is the set of entities missed by the LLM and added by experts, and *E*_*final*_ is the final, high-fidelity set of entities.

**Figure 3:**
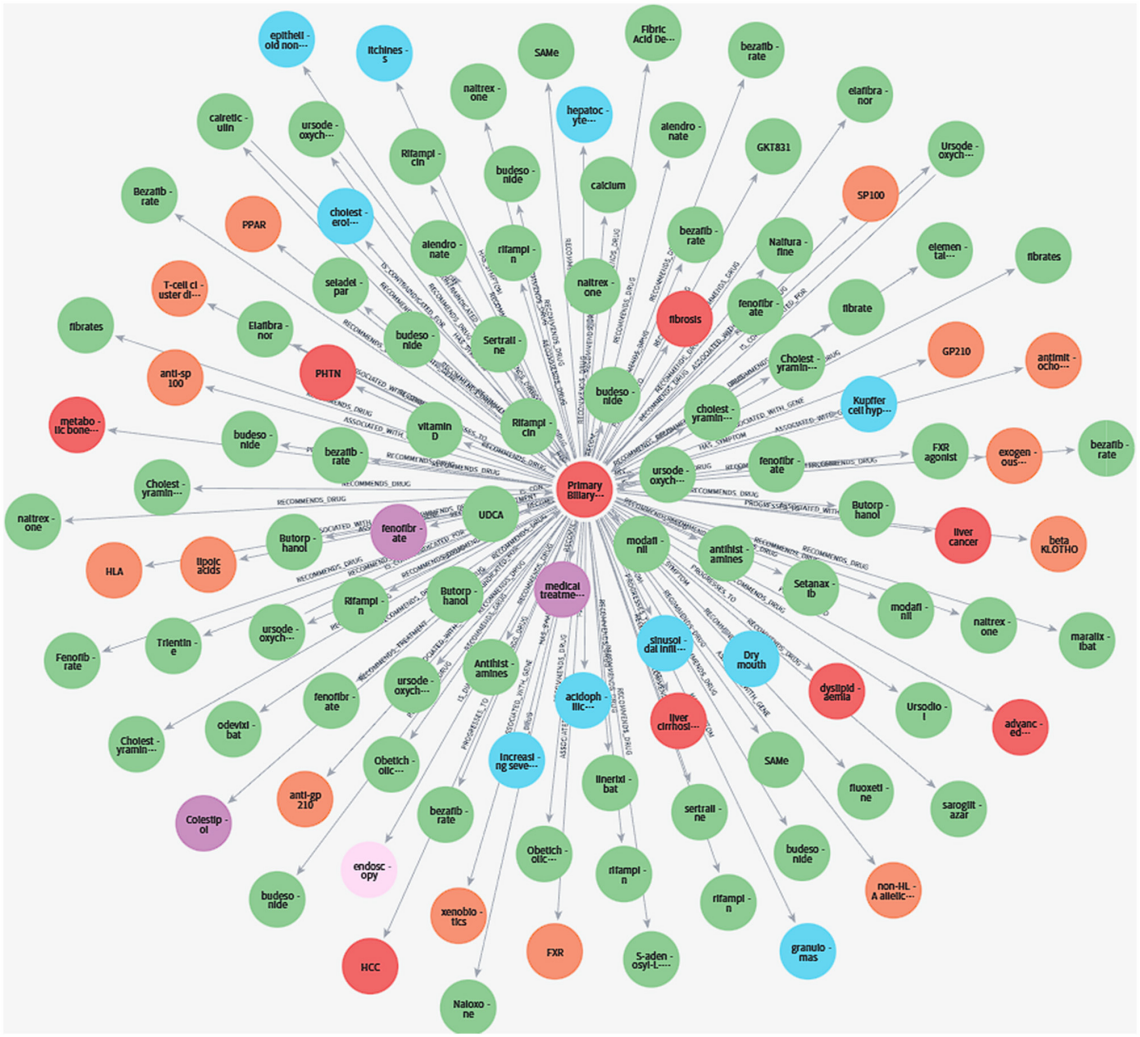
Partial visualization of the liver disease knowledge graph. This figure is a sample visualization of the liver disease knowledge graph, showing the network of interconnected medical entities and their relationships.

Finally, during the final loading and quality control stage, the high-fidelity knowledge, having been reviewed and validated by experts, is loaded and stored in a Neo4j graph database to form the definitive hepatology knowledge graph. We employ an automated quality control system that generates a quantitative evaluation report for the final graph, ensuring it adheres to pre-defined quality standards. A portion of the resulting knowledge graph is illustrated in Figure 3.

### 2.3 Design and implementation of the agent workflow

To effectively address the complexities of clinical inquiries, our framework is centered on an intelligent agent designed to meet the specific knowledge and reasoning requirements of the hepatology domain. The primary objective is to simulate the deliberative reasoning process of a human expert. To this end, we constructed an agent with autonomous decision-making capabilities, employing a dynamic, state-driven architecture. This design enables the agent to independently navigate a complete reasoning cycle of planning, execution, evaluation, and iterative correction based on the real-time state of the task, thereby significantly enhancing its ability to resolve complex clinical questions.

#### 2.3.1 Logical architecture and state-driven mechanism

**Figure 4:**
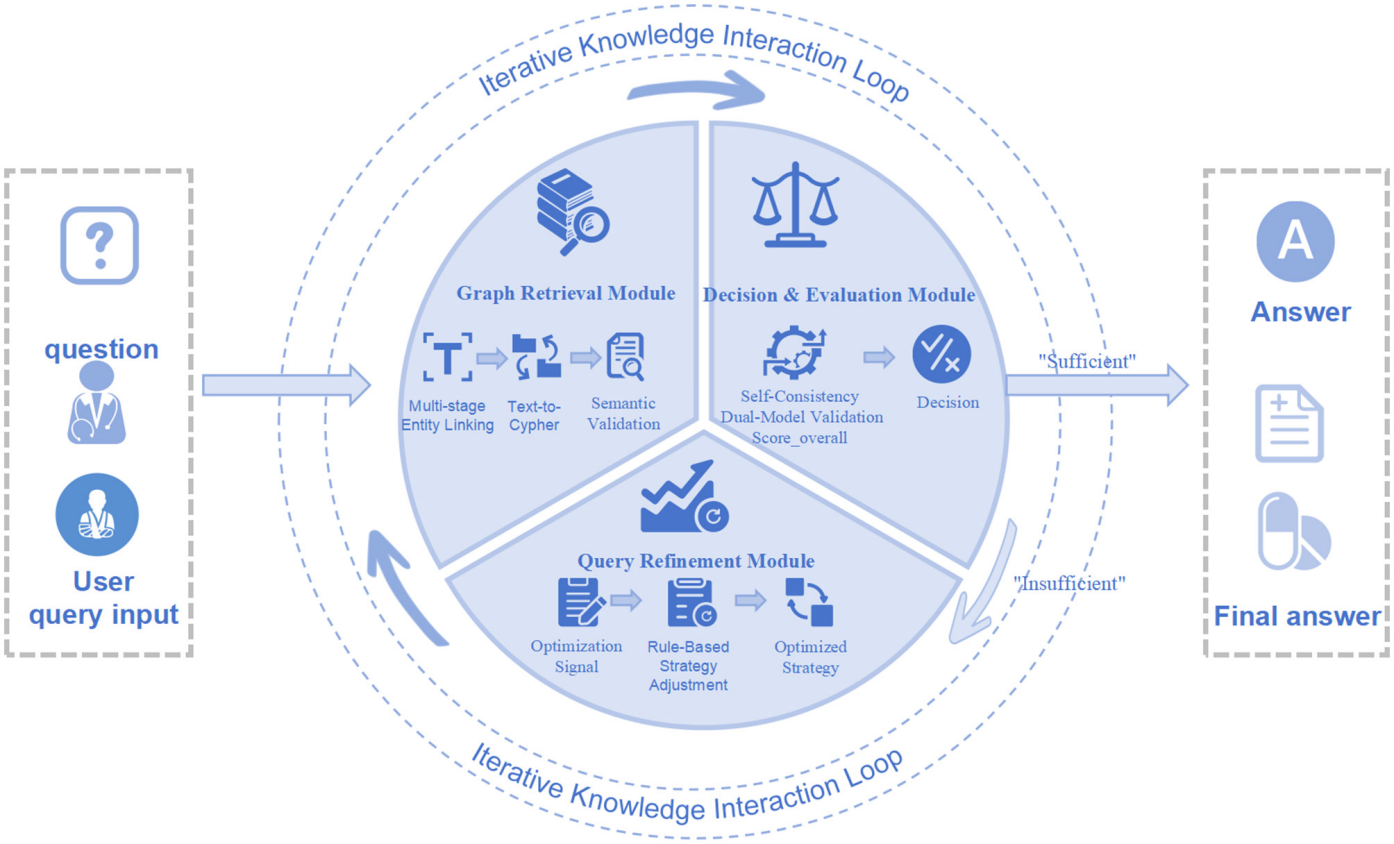
Workflow. This figure details the agent's iterative workflow, highlighting the self-correcting loop where retrieved information is evaluated and the query is refined if necessary before generating an answer.

This framework is built upon LangGraph, and its core is a dynamic reasoning architecture driven by a state machine. A centralized “graph state” object encapsulates and propagates dynamic information required throughout the problem-solving workflow, including the original clinical query, contextual evidence retrieved from the knowledge graph, intermediate queries, and the final generated answer. The “agentic” nature of this architecture is manifested in its dynamic conditional workflow. Unlike the fixed execution paths of traditional linear processes, this framework embeds autonomous decision logic at key nodes, allowing the agent to dynamically select the subsequent path based on the current state. After a user inputs a clinical question, the system first performs intent recognition and initial parsing. It then enters the graph retrieval module to generate and execute a Cypher query. After obtaining the initial evidence set, a “strategy selection node” evaluates the quality of the retrieved results. This judgment is made using dual-model validation and self-consistency methods. If this evaluation deems the evidence insufficient or ambiguous, it triggers a “self-correction loop.” This loop instructs the system to adjust its retrieval strategy, thereby altering the search path, and returns to the graph retrieval module for a new round of evidence gathering. This “retrieve-evaluate-adjust strategy” self-correction loop, combined with “dual-model-self-consistency” validation, ensures the rigor and flexibility of the reasoning process. This core workflow is depicted in Figure 4 and formalized in Algorithm 1.

Algorithm 1 Iterative query and answering framework.

Input: Initial Question Q_0_, Knowledge Graph Database KG, and Maximum Iterations T
1 Entities ← EntityLinker.link_entities(Q_initial_)
2 Cypher ← TextToCypher.convert(Q_initial_, Entities)
3 Contextcurrent ← GraphEngine.execute(Cypher, Strategy_current_)
4 A_final_ ← null
5
6 for t = 1 to T do
7 Decision ← DecisionModule.evaluate_sufficiency(Q_initial_, Context_current_)
8 if Decision.action = “USE_CURRENT” then
9 A_final_ ← AnswerGenerator.generate(Q_initial_, Context_current_)
10 break
11 else if Decision.action = “RETRY_SEARCH” then
12
13 Strategy_current_ ← StrategyOptimizer.optimize(Strategy_current_, Decision)
14 Context_current_ ← GraphEngine.execute(Cypher, Strategy_current_)
15 else
16 A_final_ ← AnswerGenerator.generate(Q_initial_, Context_current_)
17 break
18
19 if A_final_ = null then
20 if Context_current_ is not empty then
21 A_final_ ← AnswerGenerator.generate(Q_initial_, Context_current_)
22 else
23 A_final_ ← AnswerGenerator.generate_llm_fallback(Q_initial_)Output: A_final_

#### 2.3.2 Core functional modules of the agent

To ensure the framework's semantic precision and logical reasoning reliability in clinical scenarios, all core modules are built upon the gpt-4-1106-preview and GPT-3.5-Turbo models (39). These modules were implemented using a sampling temperature of T = 0.1. The function and implementation logic for each module are detailed as follows.

##### 2.3.2.1 Graph retrieval module

The Graph Retrieval Module is the starting point of the workflow, responsible for translating unstructured natural language queries into structured graph traversal paths. This process begins with a multi-stage entity linking strategy. The system first attempts an exact match against the UMLS dictionary; if this fails, it activates a fuzzy matching algorithm. This algorithm employs the Levenshtein distance to quantify the similarity between a query term and a dictionary entry, with the similarity defined as shown in Equation 2:


$${\text{S}}_{\text{fuzzy}}=1-\frac{\text{Levenshtein}\left({\text{s}}_{1},{\text{s}}_{2}\right)}{\text{max}\left(\text{len}\left({\text{s}}_{1}\right),\text{len}({\text{s}}_{2})\right)}$$ (2)

Where *s*_1_ and *s*_2_ represent the query token and the dictionary entry, respectively. An entry is considered a candidate only if its similarity score exceeds the pre-set threshold of 0.8. Subsequently, the system utilizes the Transformer-based embedding model sentence-transformers to calculate the semantic similarity between the query and the candidate entities. Candidate entities are ranked by calculating the cosine similarity between the query vector and each candidate entity vector, as shown in Equation 3:


$$\text{cos}\left(\theta \right)=\frac{{\text{V}}_{\text{q}}•{\text{V}}_{\text{c}}}{\left\|{\text{V}}_{\text{q}}\right\|\left\|{\text{V}}_{\text{c}}\right\|}$$ (3)

The final entity ranking is determined by a composite score of the fuzzy matching and semantic similarity results, followed by a disambiguation step based on the query context. After completing entity linking, the system enters the Text-to-Cypher conversion stage. This stage employs a dual-model architecture: a primary model (GPT-4) and a secondary model (GPT-3.5-Turbo) independently generate Cypher query statements. A consistency check is then performed between their outputs to mitigate the risk of LLM hallucinations. Finally, the graph execution engine retrieves the relevant subgraph based on the query intent. This process is governed by a deterministic, rule-based automated workflow with pre-defined mappings between query intent and search strategy:For fact/diagnosis/symptom-type queries, the system automatically selects Breadth-First Search (BFS) and sets a shallow depth (e.g., k = 2). For causal/multi-hop queries that require deep exploration along specific relation chains, the system automatically selects Depth-First Search (DFS) and sets a greater depth (e.g., k = 3 or k = 4). The BFS and DFS strategies are formally defined in Equations 4 and 5, respectively.

The set of retrieved nodes *N*_*BFS*_(*V*_0_, *k*) at step *k* can be defined by Equation 4:


$$\begin{array}{l} N_{BFS}\left(V_{0},k\right)=\left\{e\in E|dist\left(V_{0},e\right)\leq k\;\right\} \end{array}$$ (4)

where *dist*(*V*_0_, *e*)is the length of the shortest path from *V*_0_ to *e*.

*P*_*DFS*_(*V*_0_, *k*), at a depth of *k* is given by Equation 5:


$$\begin{array}{c} P_{DFS}\left(V_{0},k\right)\\ =\left\{\left(V_{0},V_{1},\ldots ,V_{J}\right)|V_{0}\in V_{0},\left(V_{i-1},V_{i}\right)\in R,\forall i=1..j,j\leq k\right\} \end{array}$$ (5)

Here, (*V*_*i*−1_, *V*_*i*_) denotes a relation between the entities *V*_*i*−1_ and *V*_*i*_.

##### 2.3.2.2 Decision and evaluation module

The retrieved subgraph subsequently enters the Decision Evaluation Module. This module's core task is to assess the “sufficiency” of the retrieved results, determining whether the current information is sufficient to answer the user query. The evaluation employs a dual-model cross-validation mechanism, wherein the primary and secondary models each conduct 3 independent self-consistency votes. Each model forms a preliminary decision (“Sufficient” or “Insufficient”) via majority vote and computes an internal consistency score, as shown in Equation 6:


$$\begin{array}{l} C_{internal}=\frac{max\left(N_{sufficient},N_{insufficient}\right)}{N_{total}} \end{array}$$ (6)

Concurrently, the system calculates a series of external, objective metrics, including coverage, the number of retrieved nodes, information density, the average number of attributes per node, and the critical metric of semantic relevance. Semantic relevance is computed by calculating the vector cosine similarity between the user query and the aggregated context of the retrieved subgraph. These metrics are then integrated into a comprehensive score, which is a weighted average of semantic relevance, normalized coverage, and normalized density, as shown in Equation 7:


$$\begin{array}{c} Score_{overall}\\ ={w_{sem}S}_{sem}+w_{cov}Norm\left(N_{nodes}\right)+w_{den}Norm\left(D_{avg}\right) \end{array}$$ (7)

Here, the weights,ω_*sem*_= 0.4, ω_*cov*_=0.3, ω_*den*_= 0.3 reflect the system's high emphasis on semantic accuracy. If the dual-model voting results in a discrepancy, the system relies on this *Score*_*overall*_ for adjudication: if *Score*_*overall*_>τ_*high*_, the context is deemed “Sufficient”; if *Score*_*overall*_ < τ_*low*_, it is deemed “Insufficient.”

##### 2.3.2.3 Query refinement module

If the Decision Evaluation Module deems the context “Insufficient,” the workflow triggers the Query Optimization Module. In contrast to traditional approaches that rely on LLMs to rewrite Cypher statements, our system implements a deterministic strategy optimizer. This optimizer iteratively refines the search by modifying the parameters of a search strategy object, rather than modifying the Cypher text itself. The optimizer follows a rule-based decision tree. If coverage is below its threshold and the search depth has not reached its maximum limit, the search depth is increased. If semantic relevance is below its threshold, the system switches the search strategy or increases the node limit. If information density is below its threshold, the node limit is increased. This self-correction loop iterates a maximum of three times, ensuring the query optimization process is controllable, efficient, and stable.

##### 2.3.2.4 Answer synthesis module

When the query optimization loop terminates, the workflow enters the Final Answer Generation Module. First, a Context Integrator compiles the retrieved nodes, relations, and path information into a structured textual context. Subsequently, the system employs an integrated answer generation strategy. This strategy utilizes a single call to the primary large language model to fuse the knowledge graph context with the model's internal medical knowledge, generating a unified and fluent response. Through sophisticated prompt engineering, the model is guided to use in-line citations directly, clearly attributing the source of each piece of information. Finally, the system calculates a comprehensive confidence score *C*_*final*_. This score integrates Cypher generation consistency *C*_*cipher*_, final decision confidence *C*_*decision*_, the retrieval coverage score *S*_*coverage*_, and the average entity linking confidence *C*_*entity*_, while subtracting a penalty term that increases with the number of iterations *P*_*iteration*_, as shown in Equation 8:


$$\begin{array}{c} C_{final}\\ =w_{1}C_{cypher}+w_{2}C_{decision}+w_{3}S_{coverage}+w_{4}C_{entity}-P_{iteration} \end{array}$$ (8)

Where *P*_*iteration*_ = (*k*−1) × 0.05, If the knowledge graph information is still deemed insufficient after retrieval and optimization, the system automatically falls back to using only its general parametric knowledge to answer the question. This response is appended with a clear warning message, ensuring the workflow's robustness under all conditions.

## 3 Experiments and results

### 3.1 Experimental setup

#### 3.1.1 Evaluation dataset

This study involved the construction of an evaluation dataset comprising 30 questions designed to simulate real-world clinical consultation scenarios. The dataset's development followed a rigorous validation process. One expert formulated the questions. A separate expert then authored the gold-standard answers based on clinical experience. Subsequently, another expert conducted an independent, blind validation of both the questions and answers. All disagreements were resolved through consensus. The final dataset was categorized into three distinct types, to conduct an in-depth, qualitative analysis of the behavior of our proposed agentic workflow, particularly its self-correction loop and strategy optimization.

Simple Factual Queries (10 questions): Questions targeting a single entity or its direct attributes, such as, “What condition is entecavir indicated for?”

Multi-hop Reasoning Queries (10 questions): Complex questions requiring the traversal of multiple relationships within the knowledge graph, such as, “For which etiologies of cirrhosis is liver transplantation a recommended treatment?”

Ambiguous & Colloquial Queries (10 questions): Queries that mimic informal, non-standard, or incomplete user inputs, such as, “My liver feels off and I'm bloated, what medicine should I take?”

#### 3.1.2 Baselines for comparison

To ensure a fair and relevant evaluation, all experimental models, including our proposed framework and the baselines, utilize GPT-4-1106-preview as the core foundation model. GPT-4 was selected for its state-of-the-art capabilities in natural language understanding, complex reasoning, and instruction following, which are essential for processing specialized clinical questions. Using a single, powerful foundation model allows for performance differences to be attributed directly to the architectural advantages of each framework rather than to variations in the underlying LLM.

To comprehensively evaluate our framework, we compared its performance against three representative baselines:

Standalone LLM: The standard GPT-4 model without access to any external knowledge base. This baseline measures the performance of the raw foundation model.

Standard RAG: A conventional RAG implementation that uses vector-based retrieval over a knowledge base composed of the plain text from the 53 clinical guidelines. This baseline serves to highlight the advantages of using a structured knowledge graph over unstructured text.

Basic Graph RAG (40): An ablated version of our framework that proceeds directly from graph retrieval to answer synthesis, omitting the “Decision & Evaluation” and “Query Refinement” modules. This baseline is designed to isolate and quantify the performance gains attributable to our framework's self-correction capabilities.

#### 3.1.3 Evaluation metrics

To conduct a comprehensive and objective evaluation, we employed the RAGAS framework (41), a suite designed to systematically assess the performance of RAG systems. We focused on three key metrics: Faithfulness, Context Recall, and Answer Relevancy.

Faithfulness: This metric evaluates the factual consistency of the generated answer by measuring how well it is grounded in the retrieved context. An answer is considered faithful if all the claims it makes can be inferred from the provided information. The score is calculated as the ratio of verified statements to the total number of statements in the answer, as shown in Equation 9:


$$\begin{array}{l} F=\frac{\left|V\right|}{\left|S\right|} \end{array}$$ (9)

Context Recall: This metric measures the extent to which the answer utilizes all relevant information present in the retrieved context. A high score indicates that the model has successfully incorporated all necessary ground-truth information to form a comprehensive response. It is calculated as shown in Equation 10:


$$\begin{array}{l} CR=\frac{numberofextractedsentences}{totalnumberofsentencesinc\left(q\right)} \end{array}$$ (10)

Answer Relevancy: This metric assesses how pertinent the generated answer is to the original user query. The score is calculated by first using an LLM to generate a set of potential questions from the generated answer, and then measuring the average cosine similarity between these generated questions and the original question. The formula is defined as shown in Equation 11:


$$\begin{array}{l} AR=\frac{1}{n}\overset{n}{\underset{i=1}{\sum }}sim\left(q,q_{i}\right) \end{array}$$ (11)

The LLM generates potential questions *q*_*i*_ based on the answer, and for each question *q*_*i*_, it calculates a similarity score *sim*(*q, q*_*i*_) with the original query *q*. The answer relevance of the question *q* is scored as *AR*.

To address the limitations of automated metrics in assessing the nuanced quality and safety of clinical answers, we introduced a manual expert evaluation. The two clinical hepatologists who participated in the dataset construction conducted an independent, blind review of all model-generated answers. Responses were evaluated based on Accuracy, Completeness, and Safety using a 5-point Likert scale (1 = Very Poor, 3 = Acceptable, 5 = Excellent).

### 3.2 Results

#### 3.2.1 Entity linking performance evaluation

We first conducted an independent evaluation of the multi-stage entity linker within the Graph Retrieval Module. Using a benchmark of 30 questions, we assessed performance based on precision, recall, and F1-score. This evaluation capability was implemented within our experimental code module. The results are presented in Table 3.

**Table 3:** Performance metrics of the multi-stage entity linker.

**Metric**	**Score**
Precision	0.96
Recall	0.94
F1-score	0.96

In addition to these quantitative metrics, we evaluated the system's ability to handle query variations. As shown in Table 4, the system successfully normalizes diverse phrasings, colloquialisms, and misspellings to the correct nodes. Furthermore, we conducted a failure case analysis, presented in Table 5, which demonstrates the system's handling of non-sensical terms and semantic errors via its fallback and validation modules.

**Table 4:** Examples of query variation normalization.

**Query variation**	**Normalized entity**	**Cypher snippet**
“What is hepatic steatosis?”	Hepatic steatosis -> fatty liver	…WHERE n.name = “fatty liver”…
“Tell me about liver fat”	liver fat -> fatty liver	…WHERE n.name = “fatty liver”…
“What is HCC?”	HCC -> hepatocellular carcinoma	…WHERE n.name = “Hepatocellular carcinoma”…
“How to treat cirrosis?”	Cirrosis -> cirrhosis	…WHERE n.name = “cirrhosis”…

**Table 5:** Analysis of system failure modes and edge cases.

**Failure type**	**Example**	**System response**
Linking failure	“What is ‘liver gunk'?” (non-sensical term)	Failed to link entity. The system, as designed, falls back to the llm_fallback mode and issues a warning to the user.
Semantic error	“Does cirrhosis treat hepatitis?” (logical error)	The semantic_validator.py module intercepts the semantic error, to initiate a query rewrite.

#### 3.2.2 Automated evaluation results

To evaluate the performance and competitiveness of our framework on the clinical task, we conducted a comparative analysis against the established baselines. The answers generated by each model for the evaluation dataset were quantitatively assessed using the RAGAS framework. The results of this comparison are summarized in Table 6.

**Table 6:** Comparative evaluation of model performance.

**Model**	**Faithfulness**	**Context recall**	**Answer relevancy**
GPT-4	0.82	0.74	0.70
Standard RAG	0.86	0.76	0.73
Graph RAG	0.87	0.83	0.85
Our framework	0.94	0.92	0.91

This table presents a quantitative comparison of the proposed framework's performance against three baseline models (Standalone GPT-4, Standard RAG, and Basic Graph RAG).

The experimental results demonstrate that the framework proposed in this study outperformed all baselines across the three core metrics. The superior performance in faithfulness indicates that by combining a structured knowledge graph with strict evidence constraints, the framework can substantially mitigate factual hallucinations and ensure answer accuracy. For context recall, our model's score (0.92) was also significantly higher than the baseline models, demonstrating that its self-correction loop enables more comprehensive retrieval and utilization of relevant knowledge, thereby generating more comprehensive responses. Furthermore, our model achieved the highest score (0.91) in answer relevance, showing its ability to precisely capture user intent while concurrently enhancing accuracy and completeness.

#### 3.2.3 Expert evaluation results

The expert evaluation results as shown in Table 7 further corroborate the findings of the automated evaluation and underscore the advantages of our framework for clinical applications.

**Table 7:** The expert evaluation results.

**Model**	**Accuracy**	**Completeness**	**Safety**
GPT-4	4.1	4.0	4.1
Standard RAG	4.3	4.2	4.3
Graph RAG	4.4	4.3	4.4
Our framework	4.8	4.6	4.9

The expert evaluation results demonstrate that our model achieved the highest scores across all dimensions. Experts noted that a baseline GPT-4, when faced with complex clinical questions, occasionally generated plausible but clinically risky recommendations (Safety Score: 4.2). Standard RAG and Basic Graph RAG frequently omitted key information, particularly when handling vague or multi-hop queries. In contrast, our agentic framework identifies information insufficiency via the “Decision Evaluation” module and performs iterative supplementation using the “Query Optimization” module, thereby significantly enhancing both the completeness and clinical safety of the generated answers.

## 4 Discussion

### 4.1 Advantages and limitations

The experimental results clearly demonstrate the significant advantages of the Agentic Graph RAG framework in the specialized field of hepatology. Traditional general-purpose LLMs, lacking factual grounding, exhibit high hallucination rates, failing to meet the stringent requirements of clinical applications for high precision and reliability. While Standard RAG enhances factuality, its shallow understanding of unstructured text limits its capacity for deep logical reasoning. Our framework, grounded in an expert-reviewed knowledge graph, ensures an authoritative knowledge source. This provides a solid factual anchor for reasoning and greatly enhances answer faithfulness and accuracy. Our framework's superiority stems not from the LLM itself, but from the robust agentic architecture designed to constrain it. Unlike independent LLMs, which pose a high risk of harmful hallucinations, or Standard RAG, which retrieves isolated text, our framework is anchored in an expert-validated, attribute-rich property graph. This addresses the limitations of simple triple KGs by encoding fine-grained clinical knowledge, such as conditional criteria, drug approval status, and negative relations. Furthermore, our work extends beyond basic Graph RAG by introducing a multi-stage self-correction loop. This agentic workflow prevents “syntactically correct but semantically erroneous” queries via pre-execution semantic validation. The use of self-consistency voting and dual-model cross-validation provides a highly reliable mechanism for evaluating information sufficiency. A deterministic strategy optimizer ensures that iterative refinement is stable, fast, and controllable, avoiding the unpredictability of LLM-based query rewriting. Finally, if the loop terminates without sufficient KG evidence, the system's fallback mechanism automatically appends a conspicuous disclaimer. This informs clinicians that the answer is not graph-verified, thereby mitigating the risk of using non-grounded information in high-risk decisions. Despite this promising performance, our study has limitations. First, constructing a high-fidelity knowledge graph is labor-intensive and requires significant expert intervention. Its knowledge base relies on published clinical guidelines, which may introduce a knowledge lag compared to the latest research. Second, our evaluation dataset, while meticulously curated for in-depth qualitative analysis of the agentic workflow, is modest in size. It demonstrates the framework's capabilities but is insufficient to establish broad statistical significance. Third, our chosen baselines (GPT-4, Standard RAG, Basic Graph RAG) were selected to measure the performance gains of our novel agentic architecture relative to non-agentic methods using the same foundation model. Comparison with external, state-of-the-art clinical QA systems remains a crucial next step.

### 4.2 Future work

To address the current limitations and further enhance system capabilities, we have planned the following directions for future work. First, we will benchmark this framework against external, state-of-the-art clinical QA systems to validate its performance in broader domains. Second, to overcome the static nature of the KG, we will evolve the architecture into a multi-agent collaborative system. This will involve introducing specialized agents capable of collaboratively retrieving and integrating information from other external sources, such as the latest medical literature and clinical trial databases, to address the limitations of a single knowledge graph. Third, we will implement a hybrid retrieval system that combines the precision of graph retrieval (for entity-centric queries) with the semantic breadth of vector retrieval (for more conceptual questions), thereby providing a more comprehensive context. Finally, we will explore the use of semi-autonomous agents to proactively monitor new literature, suggest knowledge updates, and integrate them into the knowledge graph following expert validation, thereby creating a dynamic and continuously evolving knowledge ecosystem.

## Data Availability

Publicly available datasets were analyzed in this study. This data can be found here: https://pubmed.ncbi.nlm.nih.gov/.
